# Prolonged Right Ventricular Outflow Tract Endocardial Activation Duration and Presence of Deceleration Zones in Patients With Idiopathic Premature Ventricular Contractions. Association With Low Voltage Areas

**DOI:** 10.3389/fphys.2021.699559

**Published:** 2021-07-02

**Authors:** Leonor Parreira, Pedro Carmo, Rita Marinheiro, Dinis Mesquita, José Farinha, Ana Esteves, Pedro Amador, António Ferreira, Marta Fonseca, Rui Caria, Pedro Adragao

**Affiliations:** ^1^Department of Cardiology, Hospital Centre of Setubal, Setubal, Portugal; ^2^Department of Cardiology, Luz Hospital Lisboa, Lisbon, Portugal

**Keywords:** premature ventricular beat, idiopathic, right ventricular outflow tract, deceleration zone, low voltage areas

## Abstract

**Background and Aims:**

The wavefront propagation velocity in the myocardium with fibrosis is characterized by the presence of deceleration zones and late activated zones, that are absent in the normal myocardium. Our aim was to study the right ventricular outflow tract (RVOT) endocardial activation duration in sinus rhythm, and assess the presence of deceleration zones, in patients with premature ventricular contractions (PVCs) and in controls.

**Methods:**

We studied 29 patients with idiopathic PVCs from the outflow tract, subjected to catheter ablation that had an activation and voltage map of the RVOT in sinus rhythm. A control group of 15 patients without PVCs that underwent ablation of supraventricular arrhythmias was also studied. RVOT endocardial activation duration and number of 10 ms isochrones across the RVOT were assessed. Propagation speed was calculated at the zone with the higher number of isochrones per cm radius. Deceleration zones were defined as zones with >3 isochrones within 1 cm radius. Low voltage areas were defined as areas with local electrogram with amplitude <1.5 mV.

**Results:**

The two groups did not differ in relation to age, gender or number of points in the map. RVOT endocardial activation duration and number of 10 ms isochrones were higher in the PVC group; 56 (41–66) ms vs. 39 (35–41) ms, *p* = 0.001 and 5 (4–8) vs. 4 (4–5), *p* = 0.001. Presence of deceleration zones and low voltage areas were more frequent in the PVC group; 20 (69%) vs. 0 (0%), *p* < 0.0001 and 21 (72%) vs. 0 (0%), *p* < 0.0001. The wavefront propagation speed was significantly lower in patients with PVCs than in the control group, 0.35 (0.27–0.40) vs. 0.63 (0.56–0.66) m/s, *p* < 0.0001. Patients with low voltage areas had longer activation duration 60 (52–67) vs. 36 (32–40) ms, *p* < 0.0001, more deceleration zones, 20 (95%) vs. 0 (0%), *p* < 0.0001, and lower wavefront propagation speed, 0.30 (0.26–0.36) vs. 0.54 (0.36–0.66) m/s, *p* = 0.002, than patients without low voltage areas.

**Conclusion:**

Right ventricular outflow tract endocardial activation duration was longer, propagation speed was lower and deceleration zones were more frequent in patients with PVCs than in controls and were associated with the presence of low voltage areas.

## Introduction

It is usually accepted that PVCs from the outflow tracts result from triggered activity and occur in structurally normal hearts (Lerman, 2015). Most studies with cardiac magnetic resonance (CMR) have failed to demonstrate the presence of structural abnormalities. Still, studies with intracardiac mapping have shown the presence of low voltage areas (LVA) (Yamashina et al., 2009; Furushima et al., 2012; Letsas et al., 2019; Parreira et al., 2019a, c, 2020). Whether these LVAs correspond to a form of incipient manifestation of a disease of the RVOT remains unproved. Also, it was previously pointed out that patients with frequent PVCs had worse RV global longitudinal strain values (and therefore sub-clinical myocardial dysfunction) than healthy controls patients with structurally apparent normal hearts, irrespective of the absence of abnormalities on the CMR (Fonseca et al., 2021). Fibrosis is associated with late local activation and areas of slow conduction and slower wavefront propagation speed, known as deceleration zones (DZ) (Raiman and Tung, 2018; Aziz et al., 2019). It has been previously observed that the velocity of the wavefront propagation of the PVC across the RVOT is slower in patients with PVCs from the RVOT than from the left ventricular outflow tract (LVOT), but the authors attributed this finding to the presence of differences in myocardial fiber orientation (Herczku et al., 2012; Masuda et al., 2018). However, this finding might be due to subtle disease of the RVOT manifested by the presence of LVAs. Based on previous studies it has become clear that activation delay is a hallmark of arrhythmogenic right ventricular cardiomyopathy (ARVC) (Tandri et al., 2009).

The aim of this study was to assess the pattern of propagation of the endocardial activation in RVOT looking for the presence of DZs in patients with PVCs and in controls and evaluate the association with the presence of LVAs.

## Materials and Methods

### Study Population

From July 2019 to December 2020, we prospectively studied consecutive patients with symptomatic idiopathic frequent PVCs (>10,000/24 h) with a LBBB or RBBB, vertical axis, negative polarity in lead aVL, that were referred for catheter ablation by the same operator. Independently of the site of origin of the arrhythmia all patients had an electroanatomical activation and voltage map of the RVOT obtained in sinus rhythm This study was carried out in two hospitals. All patients underwent transthoracic echocardiography, including 2-dimensional, M-mode, and Doppler study and 12-lead electrocardiogram (ECG). All patients in the PVC group had a CMR with late gadolinium enhancement (LGE) to exclude the presence of structural heart disease.

Arrhythmogenic right ventricular cardiomyopathy was ruled out according to the Task Force Criteria (Marcus et al., 2010). A 24-h Holter recording was performed before ablation and the number of PVCs per 24 h and the presence of episodes of non-sustained ventricular tachycardia (NSVT), defined as >3 PVCs in a run were assessed. Patients with evidence of structural heart disease, conduction delays, electrical diseases or abnormal QRS morphology, as well as patients with previous ablation were excluded.

A control group of consecutive patients without PVCs, that underwent catheter ablation of supraventricular tachycardias since 2019 and agreed to have an electroanatomical map of the RVOT obtained in sinus rhythm was also studied.

### Study Design

We assessed the duration of the endocardial activation (AD) on the RVOT in sinus rhythm as well as the presence of DZ in patients with PVCs and in controls. The presence of low voltage areas (LVAs) in sinus rhythm was also assessed. We tested for the association between the AD and DZs and the presence of LVAs.

### Electroanatomic Mapping and Ablation

Patients were studied in a fasting non-sedate state. All beta-blockers and antiarrhythmic drugs were discontinued at least five half-lives before the electrophysiological study. Diagnostic catheters were positioned via the femoral vein with fluoroscopic guidance in the His position and in the great cardiac vein via the coronary sinus. During endocardial mapping of the LVOT heparin was administrated to achieve an ACT of 250–300 s. After the completion of the sinus rhythm map, isoprenaline was administered intravenously, as needed, and titrated to a dose capable of inducing PVCs.

All patients underwent electroanatomical mapping with CARTO 3 (Biosense-Webster) or EnSite Precision (Abbott). With the former, all procedures were performed using the Niobe magnetic navigation system (Stereotaxis) working with the monoplane fluoroscopy system AXIOM Artis (Siemens) as previously described (Parreira et al., 2013). An irrigated tip Navistar RMT Thermocool catheter (Biosense-Webster) was used with a 3.5-mm distal tip electrode and a 2–5–2 interelectrode distance. With the EnSite Precision system all procedures were done manually with the monoplane fluoroscopy system BV Pulsera (Philips) and using an irrigated tip FlexAbility catheter (Abbott) with a 4-mm distal tip electrode and a 1–4–1 interelectrode distance. The ablation catheter was introduced via the femoral vein, manually advanced to the right atrium and then automatically advanced to the His bundle and RVOT in the magnetic navigation system patients or manually in the EnSite patients, under fluoroscopic guidance. The ablation catheter was then placed at multiple sites on the endocardial surface of the RVOT. The 12-lead surface ECGs and intracardiac electrograms were recorded simultaneously by a digital multichannel system, filtered at 30–300 Hz for bipolar electrograms and at 0.05–525 Hz for unipolar electrograms, displayed at 100 mm/s speed. During sinus rhythm two maps were created, activation and voltage map and during PVC, activation map.

All the intracardiac electrograms were reviewed by two senior electrophysiologists.

#### Sinus Rhythm Activation and Voltage Map

The sinus rhythm maps were obtained without isoprenaline. With the Carto system, local activation time (LAT) was defined as the time of the maximum downslope of the unipolar distal electrogram displayed on the corresponding bipolar signal. With the Ensite system LAT was defined as the time of the first peak of the bipolar electrogram (Kim et al., 2020). Total RVOT AD was measured as the time interval from the earliest RVOT endocardial activation to the latest RVOT endocardial activation recorded during sinus rhythm. The geometry of the RVOT depicting the LAT recorded was constructed in real time with the electrophysiological information color coded and superimposed on the reconstruction. The data derived from the isochronal map and analysis were obtained offline, and therefore, the procedure results were not dependent on these data. Activation isochronal maps were displayed as 10 ms isochrones, those maps were generated automatically with the Carto3 system. In the case of Ensite Precision the RVOT isochronal map was always displayed as eight 10 ms isochrones, considering the earliest isochrone the one encompassing the first 10 ms of activation time in the RVOT, and the last isochrone covering the latest activation time above 80 ms. Six other 10 ms isochrones were considered for LAT between those two extreme values. The total number of 10 ms isochrones across the RVOT was measured as well as the maximum number of 10 ms isochrones per 1 cm radius (Aziz et al., 2019). The propagation speed was assessed in the areas of the highest number of isochrones per cm radius. These areas with higher isochrone confluence per cm represent the areas with a slower conduction, and therefore, were the ones chosen to evaluate whether propagation speed was outside the accepted normal values of 0.62 ± 0.06 m/s previously described in normal myocardium (Brugada et al., 1991). The areas with abnormal wavefront propagation speed, known as DZs were defined as zones with > 3 isochrones within 1 cm radius (Aziz et al., 2019).

The electrograms were also analyzed in regard of their amplitude and the information was used to generate a 3-dimensional electroanatomical voltage map of the RVOT, with the electrophysiologic information, color-coded and superimposed on the geometry. The color display for voltage mapping ranged from purple, representing electroanatomical normal tissue (amplitude ≥ 1.5 mV), to red, representing electroanatomical scar tissue (amplitude < 0.5 mV). LVAs were defined as areas with bipolar electrograms with an amplitude < 1.5 mV. The level of RVOT/pulmonary valve junction was thoroughly determined based on electroanatomical voltage mapping by passing the catheter into the pulmonary artery and slowly withdrawing it to the RVOT. The voltage above the pulmonary valve is usually less than 0.5 mV. The area immediately below the level of the pulmonary valve displays intermediate colors, corresponding to a bipolar voltage between 0.5 and 1.5 mV, defined as the transitional-voltage zone (Yamashina et al., 2009). Presence of LVAs outside the transitional-voltage zone, were assessed.

The sinus rhythm maps were obtained in patients with PVCs and in patients from the control group.

#### PVC Activation Map and Ablation

The activation map was created by mapping several points during each PVCs while using a surface ECG lead as reference. The ablation site was selected based on the earliest endocardial activation time in relation to the onset of the surface QRS, with a QS pattern at the unipolar electrogram and confirmed by the pace mapping that provided at least 11 out of 12 pace matches between paced and spontaneous PVCs. Energy was delivered from an EP Shuttle RF generator (Stockert) between the distal electrode of the ablation catheter and a cutaneous patch, for up to 120 s, to a maximum temperature of 43°C and a power output limit of 50 W. When the application was ineffective, additional applications were delivered to sites adjacent to the earliest activation site. During ablation, light sedation with midazolam (bolus) or remifentanil (continuous perfusion) was administered when needed. Success was defined as abolition of PVCs under isoprenaline infusion until 30 min after ablation.

### Statistical Analysis

All analyses were performed using SPSS statistical software, version 26.0 (SPSS, Inc., Chicago, Illinois). Data is presented as median and lower and upper quartile (Q_1_–Q_3_) for continuous variables, as median and minimum and maximum (min-max) for ordinal variables and as absolute numbers and percentages for binary variables. Continuous variables and ordinal variables were compared with the use of Mann Whitney test for independent samples. Categorical variables were compared with the use of two-side Fischer’s exact-test or the chi square test as appropriate for independent samples. The ROC curve was used to assess the cut point value of AD that ensures the presence of LVAS and its specificity and sensitivity based on 2 × 2 contingency table and chi square test. For all tests a two-tailed *p* < 0.05 was considered as statistically significant.

### Ethics

All patients signed the informed consent form, and the study was approved by the Ethical Committee of both hospitals. The study is in compliance with the Helsinki Declaration.

## Results

### Study Population

Forty-four patients were enrolled, 29 patients in the PVC group and 15 patients in the control group of whom, twelve underwent ablation of atrioventricular nodal reentrant tachycardia, one of atrioventricular reentrant tachycardia and two of typical atrial flutter. Physical examination, 12-lead ECG and transthoracic echocardiography were normal. Both groups did not differ in relation to age, gender, or other clinical, standard electrocardiographic and echocardiographic parameters (Table 1). PVCs originated in the RVOT in 23 patients and in the LVOT in six. In the PVC group, all patients were symptomatic, all complained of palpitations and one patient had one syncopal episode typically vagal in nature. No patient had family history of sudden death. The 24-h Holter recording showed a high PVC burden with a median of 21164 (15,000–28,750) PVCs/24 h and presence of NSVT in 11 patients (38%). The CMR did not show evidence of RVOT abnormalities in any patient.

**TABLE 1:** Baseline characteristics and comparison between PVC group and control group.

	Overall sample (n = 42)	PVC group (n = 29)	Control group (n = 15)	*P* value
**Demographic data**				
Age in years, median (Q_1_–Q_3_)	53 (35–65)	51 (37–65)	56 (28–66)	0.710
Male Gender, n (%)	20 (46)	14 (48)	6 (40)	0.752
Body surface area in m^2^, median (Q_1_–Q_3_)	1.81 (1.59–1.92)	1.79 (1.57–1.95)	1.82 (1.60–1.95)	0.757
**Risk factors and medications**				
Hypertension, n (%)	17 (39)	10 (35)	7 (47)	0.521
Diabetes, n (%)	8 (18)	6 (21)	2 (13)	0.695
Betablockers, n (%)	25 (58)	18 (62)	7 (50)	0.521
Antiarrhythmic drugs, n (%)	9 (21)	8 (28)	1 (7)	0.231
**12 lead ECG**				
QRS duration in ms, median (Q1–Q3)	87 (80–90)	89 (80–90)	83 (80–90)	0.288
T wave inversion beyond V1, n (%)	0 (0)	0 (0)	0 (0)	
24-h holter monitoring*				
Number of PVCs, median (Q1-Q3)*	–	21164 (15,000–28,750)		
NSVT, n (%)*	–	11 (38)		
**Echocardiogram**				
LVEF in%, median (Q_1_–Q_3_)	58 (56–59)	57 (56–60)	58 (57–59)	0.416
LAD in mm, median (Q_1_–Q_3_)	35 (30–40)	37 (30–41)	33 (31–41)	0.121

*In the PVC group; LAD, left atrial diameter in parasternal view; LVEF, left ventricular ejection fraction; NSVT, non-sustained ventricular tachycardia; PVC, premature ventricular contractions.

### Electroanatomic Mapping and Ablation

#### Activation and Voltage Map in Sinus Rhythm

**FIGURE 1:**
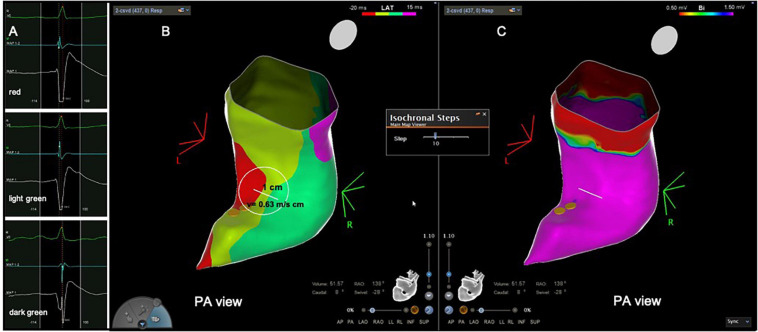
Patient from the control group with normal activation and normal voltage. **(A)** Intracardiac electrograms corresponding to the respective isochrones presented in the isochronal map. **(B)** RVOT isochronal map in sinus rhythm without DZ. **(C)** Voltage map in sinus rhythm without LVAs. Yellow dots in **(C)** His bundle tagging. DZ, deceleration zone; LVA, low voltage area; RVOT, right ventricular outflow tract.

**FIGURE 2:**
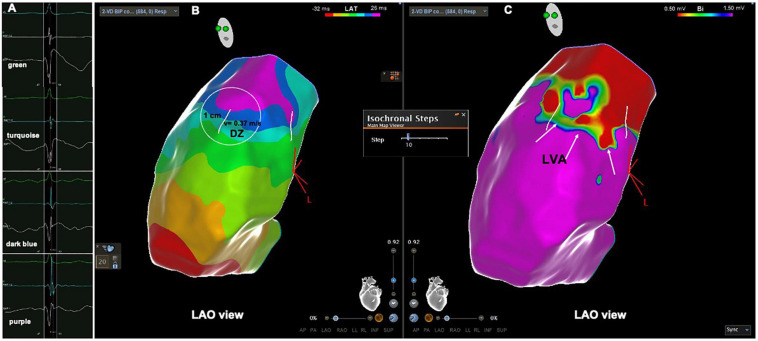
Patient from the PVC group. **(A)** Intracardiac electrograms corresponding to the respective isochrones presented in the isochronal map. **(B)** RVOT isochronal map in sinus rhythm with DZ in the RVOT free wall. **(C)** Voltage map in sinus rhythm with LVA at the same location. DZ, deceleration zone; LVA, low voltage area; RVOT, right ventricular outflow tract.

The electroanatomical mapping in sinus rhythm was successfully acquired in all patients, the median number of points per patient, collected in the RVOT to obtain the map was 387 (340–506) and was not significantly different between patients with PVCs and the control group, respectively 412 (343–533) vs. 345 (339–450), *p* = 0.193 (Table 2). The earliest RVOT endocardial activation occurred anteriorly in the free wall, while the latest endocardial activation was observed at the sub-pulmonary valve areas in all cases. The median AD, the median number of 10 ms isochrones and the maximum number of 10 ms isochrones per 1 cm radius was significantly higher in the PVC group than in the control group, respectively, 56 (41–66) vs. 39 (35–41) ms, *p* = 0.001; 5 (4–8) vs. 4 (4–5), *p* = 0.001; 4 (3–6) vs. 3 (3–3), *p* < 0.0001 (Table 2). The maximum number of 10 ms isochrones per 1 cm radius observed in the control patients was three (Figure 1). DZs were present in 20 patients (69%) and LVAs in 21 patients (72%) in the PVC group (Figure 2), but both were absent in control subjects, *p* < 0.0001. The propagation speed at the areas of confluence of 10 ms isochrones was significantly slower in patients with PVCs than in controls, 0.35 (0.27–0.40) vs. 0.63 (0.56–0.66) m/s, *p* < 0.0001.

**TABLE 2:** Sinus rhythm activation and voltage mapping data.

	Overall sample (n = 44)	PVC group (n = 29)	Control group (n = 15)	*P*-value
Number of points in the map, median (Q_1_–Q_3_)	387 (340–506)	412 (343–533)	345 (339–459)	0.193
RVOT activation duration in ms, median (Q_1_–Q_3_)	42 (37–60)	56 (41–66)	39 (35–41)	0.001
N° of 10 ms isochrones in the RVOT, median (min-max)	5 (4–8)	5 (4–8)	4 (4–5)	0.001
Max. n° of 10 ms isochrones per 1 cm radius, median (min-max)	3 (3–6)	4 (3–6)	3 (3–3)	<0.0001
Propagation speed* in m/s, median (Q_1_–Q_3_)	0.40 (0.29–0.65)	0.35 (0.27–0.40)	0.63 (0.56–0.66)	<0.0001
Presence of DZs, n (%)	20 (46)	20 (69)	0 (0)	<0.0001
Presence of LVAs, n (%)	21 (48)	21 (72)	0 (0)	<0.0001

*At the 10 ms isochronal confluence zone; DZ, deceleration zone; RVOT, right ventricular outflow tract; LVA, low voltage area; PVC, premature ventricular contractions.

#### Association Between Duration of Endocardial Activation in the RVOT and Presence of LVAs in Patients With PVCs

In the PVC group, the presence of LVAs was more frequent in patients with PVCs from the RVOT than in patients with PVCs from the left side, 83 vs. 33%, *p* = 0.033.

**FIGURE 3:**
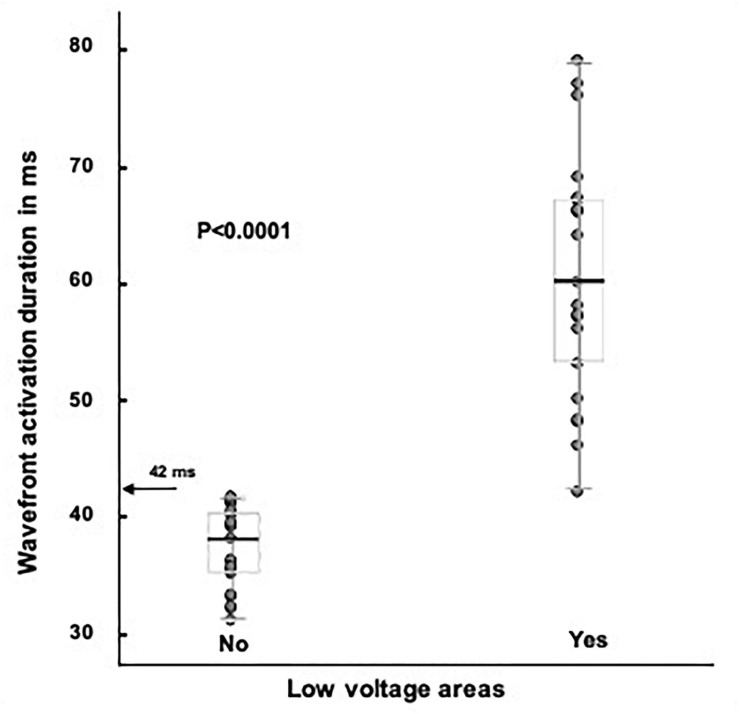
Total RVOT endocardial activation time is significantly higher in patients with LVAs, *p* < 0.0001. The value of 42 ms was calculated with the ROC curve (*p* < 0.0001). None of the patients without LVAs had AD longer than 42 ms (arrow). LVA, low voltage area; RVOT, right ventricular outflow tract.

**FIGURE 4:**
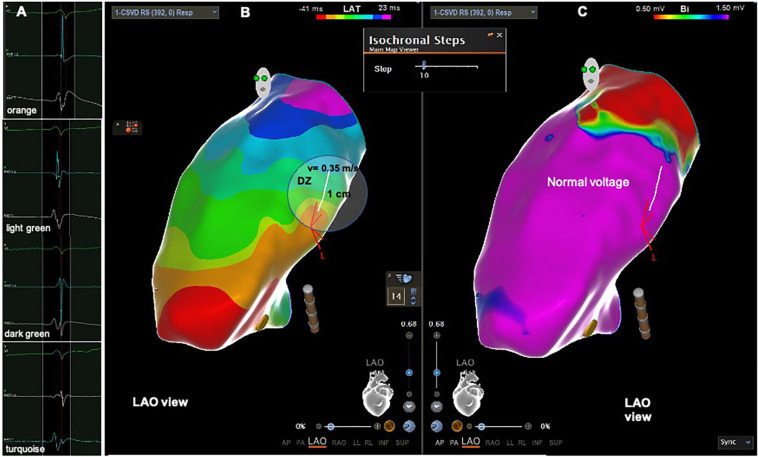
Patient from the PVC group with DZ outside the LVAs. **(A)** Intracardiac electrograms corresponding to the respective isochrones presented in the isochronal map. **(B)** RVOT isochronal map in sinus rhythm with DZ in the RVOT free wall. **(C)** Voltage map in sinus rhythm with normal voltage at the DZ. DZ, deceleration zone; LVA, low voltage area; RVOT, right ventricular outflow tract.

**FIGURE 5:**
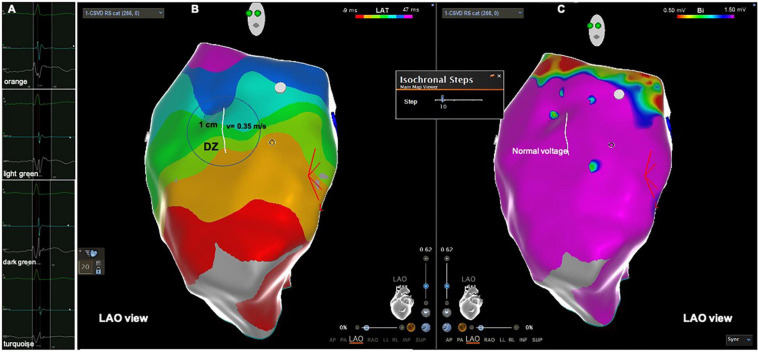
Patient from the PVC group with DZ outside the LVAs. **(A)** Intracardiac electrograms corresponding to the respective isochrones presented in the isochronal map. **(B)** RVOT isochronal map in sinus rhythm with DZ in the RVOT free wall. **(C)** Voltage map in sinus rhythm with normal voltage at the DZ. DZ, deceleration zone; LVA, low voltage area; RVOT, right ventricular outflow tract.

The longer the AD the higher the likelihood of displaying LVAs in the RVOT. According to the ROC curve the cut point value is above 42 ms, with a sensitivity and specificity of 100%, *p* < 0.0001. Patients with LVAs had a longer AD than patients without LVAS, 60 (52–67) ms vs. 36 (32–40) ms, *p* < 0.0001. All patients with LVAs had ADs above 42 ms (Figure 3), they also displayed a higher number of 10 ms isochrones across the RVOT and maximum number of 10 ms isochrones per 1 cm radius (Table 3). In twenty out of twenty-one (95%) was possible to identify the presence of DZs that were absent in all patients without LVAs, *p* < 0.0001. DZs occurred in areas of normal voltage in just two patient (10%) Figures 4, 5. The propagation speed at the zone with the higher number of 10 ms isochrones was also slower in patients with LVAs than in those without, 0.30 (0.26–0.36) vs. 0.54 (0.36–0.66) m/s, *p* = 0.002.

**TABLE 3:** Sinus rhythm activation data in the PVC group according to the presence of LVAs.

	Overall PVC Group (n = 29)	With LVAs (n = 21)	Without LVAs (n = 8)	*P*-value
Number of points in the map, median (Q_1_–Q_3_)	412 (343–533)	465 (366–533)	313 (214–544)	0.083
EAS in the RVOT, n (%)	23 (79)	19 (91)	4 (50)	0.033
RVOT activation duration in ms, median (Q_1_–Q_3_)	56 (41–66)	60 (52–67)	36 (32–40)	<0.0001
N°of 10 ms isochrones in the RVOT, median (min-max)	5 (4–8)	6 (5–8)	4 (4–5)	<0.0001
Max. n° of 10 ms isochrones per 1 cm radius, median (min-max)	4 (3–6)	4 (3–6)	3 (3–3)	<0.0001
Propagation speed* in m/s, median (Q_1_–Q_3_)	0.40 (0.29–0.65)	0.30 (0.26–0.36)	0.54 (0.36–0.66)	0.002
Presence of DZ, n (%)	20 (69)	20 (95)	0 (0)	<0.0001

*At the 10 ms isochronal confluence zone; EAS, earliest activation site; DZ, deceleration zone; LVA, low voltage area; RVOT, right ventricular outflow tract; PVC, premature ventricular contractions.

We found no association between the PVC burden and the presence of LVAs or DZ. The median number of PVCs in patients with LVAs was 21000 (13,887–28,750)/24 h vs. 23717 (18,196–29,375)/24 h in patients without LVAs, *p* = 0.301. The median number of PVCs in patients with DZs was 19082 (13,330–28,750)/24 h vs. 23134 (18,797–31,470)/24 h in patients without DZs, *p* = 0.295.

#### PVC Activation Map and Ablation

**FIGURE 6:**
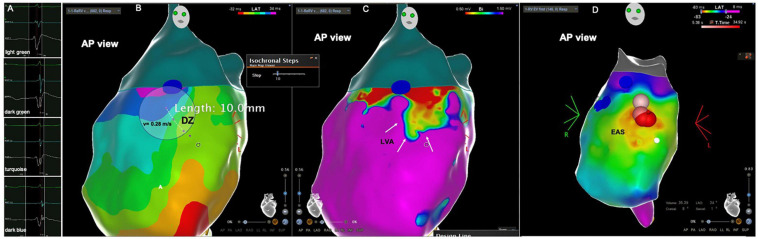
Patient from the PVC group with DZ at a LVA and the earliest activation site located in that zone. **(A)** Intracardiac electrograms corresponding to the respective isochrones presented in the isochronal map. **(B)** RVOT isochronal map in sinus rhythm with DZ in the RVOT free wall. **(C)** voltage map in sinus rhythm with LVA in the RVOT free wall. **(D)** Activation map during PVC showing the EAS at the DZ and LVA. Red and pink balls corresponding to radiofrequency application points. DZ, deceleration zone; EAS, earliest activation site; LVA, low voltage area; PVC, premature ventricular contractions; RVOT, right ventricular outflow tract.

**FIGURE 7:**
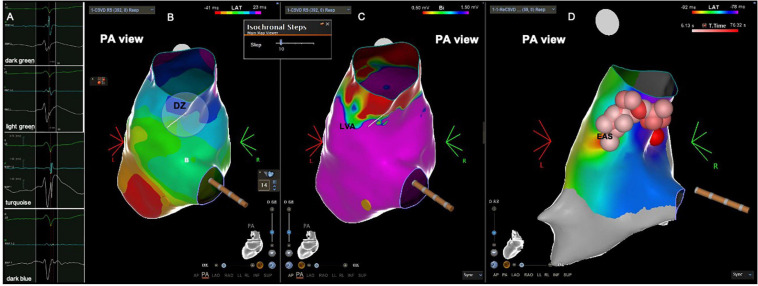
Patient from the PVC group with DZ at a LVA and the earliest activation site located in that zone. **(A)** Intracardiac electrograms corresponding to the respective isochrones presented in the isochronal map. **(B)** RVOT isochronal map in sinus rhythm with DZ in the RVOT posterior wall. **(C)** Voltage map in sinus rhythm with LVA in the RVOT posterior wall. **(D)** Activation map during PVC showing the EAS at the DZ and LVA. Red and pink balls corresponding to radiofrequency application points. DZ, deceleration zone; EAS, earliest activation site; LVA, low voltage area; PVC, premature ventricular contractions; RVOT, right ventricular outflow tract.

**FIGURE 8:**
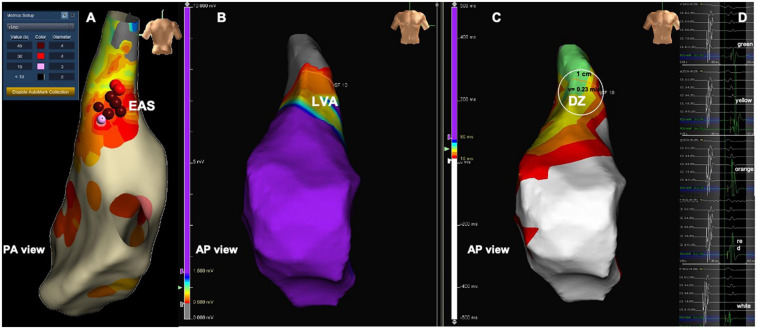
Patient from the PVC group with DZ at a LVA but the earliest activation site located outside the DZ. **(A)** Activation map during PVC showing the EAS in the RVOT posterior wall. **(B)** Voltage map in sinus rhythm with LVA in the RVOT anterior wall. **(C)** RVOT isochronal map in sinus rhythm with DZ in the RVOT anterior wall. **(D)** Intracardiac electrograms corresponding to the respective isochrones presented in the isochronal map. Red and pink balls in **(A)** corresponding to radiofrequency application points. DZ, deceleration zone; EAS, earliest activation site; LVA, low voltage area; PVC, premature ventricular contractions; RVOT, right ventricular outflow tract.

**FIGURE 9:**
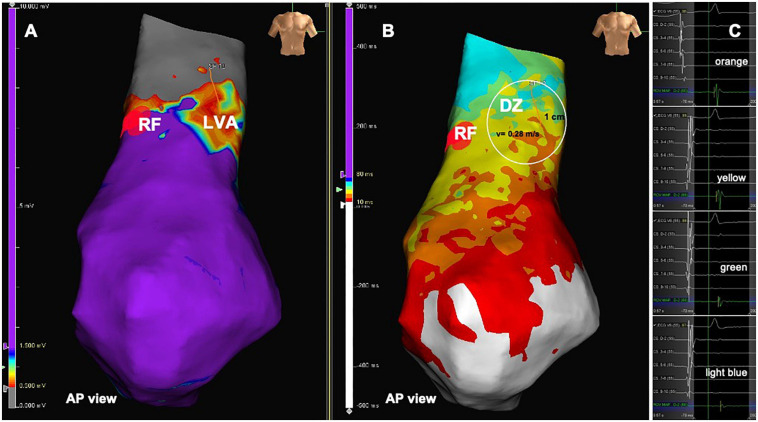
Patient from the PVC group with DZ at a LVA but the earliest activation site located outside the DZ. **(A)** voltage map in sinus rhythm with LVA in the RVOT free, red dots at site of origin of the PVCs. **(B)** RVOT isochronal map in sinus rhythm with DZ in the RVOT free wall, red dots at the site of origin of the PVCs outside the DZ. **(C)** Intracardiac electrograms corresponding to the respective isochrones presented in the isochronal map. Red dots in **(A,B)** corresponding to radiofrequency applications. DZ, deceleration zone; EAS, earliest activation site; LVA, low voltage area; PVC, premature ventricular contractions; RF, radiofrequency applications; RVOT, right ventricular outflow tract.

In the PVC group the electroanatomical mapping was performed with Carto 3 in 11 patients and with Ensite Precision in 18 patients. The earliest activation site was located in the RVOT septum in 15 patients, the RVOT free wall in 8 patients, the left coronary cusp in two, the LVOT in three and the LV summit in one. The access to the LVOT was obtained by retrograde transaortic approach in all six patients. The earliest activation site was located within the LVAs in 15 (52%) patients. This percentage increased to 71% in the group with LVAs. The earliest activation site was located in a DZ in 10 (35%) patients (Figures 6, 7), that displayed low voltage in 70% of cases (Table 4), and outside a DZ in 19 (65%) patients (Figures 8, 9). At the earliest activation site, the precocity of the LAT in relation to the onset of the surface QRS was −41 [−33–(–51)] ms. The median procedure time, fluoroscopy time and RF time were, respectively, 120 (90–130) min, 6 (4–11) min and 440 (223–648) s. The comparison between patients with DZ and patients without DZ is presented in Table 4. Success was achieved in 25 patients (86%) and was not associated with the presence of LVAs or DZs.

**TABLE 4:** Activation mapping during PVCs and ablation data according to the presence of DZs.

	PVC group (n = 29)	With DZ (n = 20)	Without DZ (n = 9)	*p*-value
Carto/Ensite, n (%)/n (%)	11 (38)/18 (62)	7 (35)/13 (65)	4 (44)/5 (56)	0.694
PVCs from RVOT/LVOT, n (%)/n (%)	23 (79)/6 (21)	18 (90)/2 (10)	4 (44)/5 (56)	0.056
Presence of LVAs	21 (72)	20 (100)	1 (11)	<0.0001
EAS in LVAs, n (%)	15 (52)	14 (70)	1 (11)	0.005
LAT at the EAS in ms, median (Q_1_−Q_3_)	−41 [−33–(−51)]	−42 [−35–(−50)]	−37 [−31–(−53)]	0.900
Procedure time in min, median (Q_1_–Q_3_)	120 (90–130)	120 (88–145)	120 (90–125)	0.934
Fluoroscopy time in min, median (Q_1_–Q_3_)	6 (4–11)	5 (4–10)	8 (4–12)	0.637
RF time in min, median (Q_1_-Q_3_)	440 (223–648)	430 (270–612)	565 (157–690)	0.760
Success, n (%)	25 (86)	17 (85)	8 (89)	1.000

*EAS, earliest activation site; DZ, deceleration zone; LAT, local activation time; LVA, low voltage area; LVOT, left ventricular outflow tract; RF, radiofrequency; RVOT, right ventricular outflow tract; PVC, premature ventricular contractions.*

## Discussion

The first important finding of this study was the demonstration of a longer duration of the endocardial activation across the endocardium of the RVOT in patients with PVCs in comparison with the control subjects. To the best of our knowledge this is the first report assessing the propagation of the wavefront of endocardial activation in sinus rhythm in patients with PVCs from the RVOT in comparison with a control group without PVCs. In our group of patients, although the AD was significantly longer in the PVC group than in controls, it was nevertheless still within the normal range previously described by other authors (Tandri et al., 2009; Letsas et al., 2018). Tandri et al. (2009) studied a group of 25 patients with frequent left bundle branch block morphology premature ventricular complexes, 14 with ARVC and 11 with idiopathic PVCs. The authors evaluated the total right ventricular endocardial activation duration and observed that an AD higher than 65 ms was highly suggestive of ARVD and was never present in patients with idiopathic PVCs. The authors concluded that total right ventricular AD was a more sensible and earlier marker of disease than low voltage. Letsas et al. (2019) performed a high-density map of the RVOT in 8 patients with Brugada Syndrome and 20 patients with idiopathic PVCs. The mean RVOT endocardial activation time was significantly prolonged in patients with Brugada Syndrome in comparison with patients with idiopathic arrhythmias, 86.4 ± 16.5 vs. 63.4 ± 9.7 ms, *p* < 0.001. None of the above-mentioned authors have studied a population of normal subjects without PVCs, therefore, the value of 65 and 63.4 ms reported as normal might as well be augmented in relation to people without PVCs.

In a previous work using non-invasive mapping we observed the presence of abnormal electrophysiological characteristics in the RVOT in a group of seven patients with PVCs from the RVOT when compared with a control group. Although we could not demonstrate the presence of an activation delay, we found the presence of a higher dispersion of the activation recovery interval across the epicardium of the RVOT in patients with PVCs when compared to normal controls, suggesting once again the presence of an underlying substrate (Parreira et al., 2019b).

The second important finding of our study was the presence of DZs in the RVOT of patients with PVCs that were absent in the control group. Also, the median activation speed at the areas with a higher confluence of isochrones, was significantly slower in the PVC group than in controls. The activation speed was particularly reduced at the zones with more than 3 isochrones per 1 cm radius (DZs) where it was half the previously accepted normal velocity of wavefront propagation (Brugada et al., 1991). The presence of DZs were associated with the presence of LVAs and were absent in patients without LVAs. Furthermore, the observed lower speed of the wavefront propagation, was worse in patients with PVCs that presented with LVAs than in those without LVAs. However, in 10% of cases the DZs occurred outside the LVAs and this was also described by Tandri et al. (2009) that observed that the presence of delayed activated areas was not always associated with decreased endocardial voltage and/or fractionated potentials. Even in the case of patients with structural heart disease as described by Aziz et al. (2019) DZ were present also in normal voltage zones. Voltage mapping is dependent on contact force, electrode area and orientation of the catheter tip in relation to the direction of the wavefront activation and so may be less accurate to evaluate substrate, whereas activation mapping is less dependent on these parameters (Aziz et al., 2019). But even the latter, is dependent on the methodology of LAT annotation and may be influenced by the presence of low amplitude pre-potentials, presence of multiple components or fractionated electrograms (Saba and Li, 2019). Nevertheless, the absence of those findings in the control group speak against the hypothesis that the slower velocity and the DZ might be due to inaccurate measurements, or to the presence of the previously described anisotropic conduction in RVOT (Brugada et al., 1991).

Masuda et al. (2018) studied the velocity of propagation of the PVC in a group of 23 patients with idiopathic arrhythmias from the outflow tracts. Those authors assessed the propagation speed indirectly by measuring the areas encompassing the first 5, 10, 15, and 20 ms isochrones. Mapping of the RVOT during the PVC was performed using an ultra-high resolution electroanatomic mapping system. The wavefront propagation speed of the PVC over the RVOT was slower for PVCs originating from the RVOT than for PVCs originating from adjacent sites. The authors used this information to differentiate arrhythmias originating from the RVOT from the non-RVOT arrhythmias as previously described by Herczku et al. (2012) using a conventional mapping catheter in a point-by-point manner. Both groups explained their findings based on the myocardial fiber arrangement in the RVOT that leads to a different propagation speed according to the site of origin of the arrhythmia. Therefore, when the PVC originates in the LVOT it reaches the RVOT through multiple connections and the activation becomes faster in this case.

On the contrary, we believe that this slower conduction is related to the presence of a pathological substrate in the RVOT rather than a difference in the connections between the origin of the arrhythmia and the RVOT. In fact, in our study group the presence of a longer AD was significantly associated with the presence of LVAs. Previous studies have shown the presence of LVAs in the RVOT of patients undergoing catheter ablation of frequent PVCs despite normal CMR (Yamashina et al., 2009; Furushima et al., 2012; Letsas et al., 2019; Parreira et al., 2019a, c, 2020). The presence of LVAs did not match the results of the CMR in any of the studies. So, it may seem difficult to support that the activation delay represented by DZs is secondary to local fibrosis. However, very subtle interstitial fibrosis cannot be completely excluded, and our findings may suggest the presence of an underlying substrate too subtle to be identified by CMR techniques. In fact, the type of interstitial fibrosis that occurs in the initial phases of ARVC is not reliably detected with CMR with LGE (Puntmann et al., 2016). Using two-dimensional speckle tracking echocardiography (2D-STE) Fonseca et al. (2021) in a previous study have demonstrated that the right ventricle global longitudinal strain was significantly lower in patients with RVOT PVCs when compared with a control group, irrespective of the previous PVCs burden and success of the catheter ablation procedure. Concerning strain values determined by CMR, Zghaib et al. (2018) have previously demonstrated, in ARVC patients, that lower regional strain could identify low voltage areas on endocardial and epicardial electroanatomical mapping and had a better correlation with VT substrate than late gadolinium enhancement sites. These subtle morphological abnormalities may be the cause for the abnormal wavefront propagation observed in our population. These LVAs have been suggested as a target for ablation in case of low PVC burden during the procedure (Wang et al., 2016; Letsas et al., 2019). The way these findings might explain the occurrence of the PVCs is controverse. Usually, conduction delay and deceleration zones are associated with re-entry (Aziz et al., 2019) and idiopathic PVCs are considered to be due to a triggered mechanism. However, we can speculate that not all patients with PVCs from the outflow tracts share the same mechanism. Probably some of them, maybe a minority are truly idiopathic with a triggered mechanism, but the majority display these conduction and voltage abnormalities that can be viewed as potential candidates for ablation. Wang et al. (2016) have shown that the SOO is located in low-voltage areas (amplitude < 0.5 mV) in 3% of patients, in transitional voltage zone (amplitude between 0.5 and 1.5 mV) in 89% of patients, and in high-voltage areas (amplitude > 1.5 mV) in 8% of patients. Likewise, Letsas et al. (2019) identified LVAs mainly located below or at the level of pulmonary valve in 39 of 44 patients and in 5 patients the voltage at the successful ablation site was normal.

In line with these previous studies, we have also noted that the earliest activation site was found to be located outside the DZ and LVAs in half the patients. Thus, it is difficult to accept that the DZ and LVAs are responsible for the PVCs in all cases. A second hypothesis to explain the presence of LVAs and DZs is that they are not the cause of the PVCs but rather the result of the electric remodeling triggered by the frequent PVCs. The median PVC burden in our group was 21164 PVC/24 h PVC. A higher PVC burden can be associated with an excess mortality in idiopathic patients (Parreira et al., 2021b), and a burden over 20,000 PVCs/24 h is associated with a higher risk of PVC-related cardiomyopathy (Nielsen et al., 2020). Fibrosis leads to conduction abnormalities and it may be the substrate for the presence of DZs, delayed activation and slower conduction, and these abnormalities may be a consequence of fibrosis and not implicated in the genesis of the PVCs. So, it remains to be demonstrated if the delayed activation and presence of DZs may denote the presence of a subtle substrate for the PVCs or the electrical and anatomical remodeling as a consequence of the high PVC burden. One way or the other, those patients should continue to be closely followed after ablatio to evaluate the evolution of the disease.

## Limitations

Two mapping systems were used with different annotation methodologies. However, assuming that the methodology is consistent for all mapping points acquired with each system, the activation duration and the presence of DZ representing intervals rather than absolute values are perfectly comparable. Thus, although the minimal and maximal absolute values may be different between systems in absolute terms, the AD and 10 ms isochrones are independent of the annotation methodology.

Another limitation is the definition of DZs as more than three isochrones per 1 cm radius. This value was based on previous studies in patients with structural heart disease and may underestimate the presence of slow conduction in patients with idiopathic PVCs. However, in our group none of the normal subjects had less than three isochrones per 1 cm radius so considering abnormal a number above three seems adequate.

Finally, it would be interesting to investigate whether these abnormalities persist after ablation of the PVCs however, using invasive electroanatomical mapping to achieve that goal would be ethical unacceptable, leaving space for other techniques like for instance non-invasive mapping, echocardiographic longitudinal strain and CMR.

## Conclusion

Patients with PVCs display a longer duration of the right ventricular outflow tract endocardial activation than controls, with presence of deceleration zones corresponding to zones with abnormal activation speed. Deceleration zones are present exclusively in patients with PVCs and occur mostly at LVAs. However, only half the patients present the SOO of the PVCs in those zones.

## Data Availability Statement

The raw data supporting the conclusions of this article will be made available by the authors, without undue reservation.

## Ethics Statement

The studies involving human participants were reviewed and approved by the Hospital Luz Ethical Committee and Hospital Center of Setubal Ethical Committee. The patients/participants provided their written informed consent to participate in this study.

## Author Contributions

LP: conceptualization, methodology, and writing. LP, PC, RM, DM, JF, AE, PAm, AF, and MF: investigation. RM, DM, RC, and PAm: reviewing. All authors contributed to the article and approved the submitted version.

## Conflict of Interest

The authors declare that the research was conducted in the absence of any commercial or financial relationships that could be construed as a potential conflict of interest.
